# *Notes from the Field:* Wastewater Surveillance for Measles Virus During a Measles Outbreak — Colorado, August 2025

**DOI:** 10.15585/mmwr.mm7502a2

**Published:** 2026-01-15

**Authors:** Grace M. Jensen, Cyrus Gidfar, Kirsten Weisbeck, Meghan Barnes, Erin Minnerath, Shannon Matzinger, Allison Wheeler

**Affiliations:** ^1^Colorado Department of Public Health and Environment; ^2^Mesa County Public Health, Grand Junction, Colorado.

**Summary**

**What is already known about this topic?**

Measles is a highly infectious vaccine-preventable disease. Measles virus is shed into wastewater, and detection of the virus can precede reporting of cases, serving as an early warning of community transmission.

**What is added by this report?**

During August 4–6, 2025, measles virus was detected in wastewater samples from a wastewater treatment plant in Mesa County, Colorado. During the next week, two measles cases were reported among residents of the area served by the same treatment plant. Detection of measles virus in wastewater with subsequent reporting of measles cases in the same area facilitated coordinated and comprehensive messaging to health care providers, encouraging them to continue recommending measles vaccination.

**What are the implications for public health practice?**

Wastewater surveillance testing for measles can alert public health authorities to possible local measles transmission before and during a measles outbreak and help guide public health preparedness and response. Wastewater surveillance is conducted for both wild-type (highly contagious) and vaccine-derived measles (weakened strain in the measles, mumps, and rubella vaccine).

Measles, a highly transmissible vaccine-preventable respiratory virus, can cause severe illness and result in hospitalization or death.* During March–July 2025, Colorado reported 16 confirmed measles cases while measles outbreaks were occurring in neighboring New Mexico, Texas, and Utah (*1*); the first five Colorado cases were confirmed by late April. Measles virus RNA shed in feces and urine can be detected in wastewater (*2*), and sequencing of the nucleoprotein gene can identify the wild-typemeasleslineagegenotype. Detection of measles virus in wastewater can precede clinical case reporting (*3*), and evidence of the value of supplementing clinical case reporting with wastewater surveillance is growing (*4*). On May 1, wastewater surveillance testing for measles virus was implemented in Colorado. In early August, the Colorado Department of Public Health and Environment (CDPHE) identified measles virus in a wastewater sample from a Mesa County wastewater treatment plant, providing local public health agency authorities with an early indicator of possible community transmission. During the next 4 days, two measles cases were reported among residents served by the same wastewater treatment plant where measles virus detection had occurred. This report describes the detection of measles virus through wastewater surveillance in Mesa County and its contribution to the subsequent outbreak response.

## Investigation and Outcomes

### Colorado Wastewater Surveillance Program

The Colorado wastewater surveillance program wasestablishedin2020inresponsetotheCOVID-19pandemic. The wastewater surveillance program monitors wastewater statewide for respiratory viruses and emerging pathogens using digital polymerase chain reaction (dPCR).^†^ On May 1, 2025, after the identification of five measles cases in the state, the wastewater surveillance program initiated a measles surveillance pilot project to supplement statewide clinical measles surveillance. Sampling occurs twice weekly at 21 sentinel wastewater treatment facilities across the state.

### Detection of Measles Virus from the Measles Wastewater Pilot Project

Between May 1, 2025, when wastewater surveillance was implemented, and August 4, 2025, no wild-type measles was detected in wastewater in Mesa County. On August 9, 2025, the state laboratory identified low-level measles detection^§^ in a sample collected on August 4 at a treatment plant serving 90,000 residents in Mesa County (Figure).

**FIGURE:**
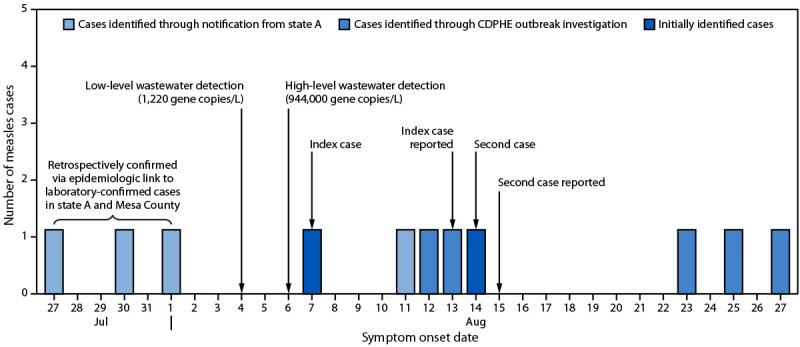
Measles symptom onset and report dates and measles wastewater detection dates* — Mesa County, Colorado, July–August 2025 **Abbreviation: **Colorado Department of Public Health and Environment. * The low-level detection in a sample collected on August 4 was reported on August 9, and the high-level detection in a sample collected on August 6 was reported on August 11.

At that time, no measles cases had been reported among residents of Mesa County during 2025. A second wastewater sample collected on August 6 with results received on August 11 had the highest concentration of measles virus RNA (944,000 gene copies per liter) since the pilot began in May; genomic sequencing after dPCR confirmed measles virus genotype D8. The sharp increase in measles virus concentration identified between the two sequential samples suggested community transmission (*1*). The local public health agency was notified the same day and coordinated with local and state public health officials, including planning for increased staffing to support an outbreak response in the event of a confirmed measles case in the area. Delivery of wastewater samples collected on August 11 and August 13 was delayed at the laboratory because of awildfire; these samples were not in the acceptable temperature range for testing and were considered invalid. Measles virus was not detected in the next sample, which was collected on August 18. This activity was considered public health surveillance and exempt from human subjects review by CDPHE.

### Identification of Measles Cases in the Region of the Affected Sewershed

On August 13, 2 days after the high-concentration measles virus detection was reported, the local public health agency was notified of a suspected measles case in an unvaccinated patient aged 10–19 years. This index patient had spent time in the area served by the sewershed while infectious (i.e., from 4 days before through 4 days after rash onset) and had symptom onset on August 7. A second case, in an unvaccinated patient aged 10–19 years who worked with the index patient, was identified in the same region on August 15, 1 day after symptom onset. Neither patient had traveled outside the immediate area, and neither reported a known measles exposure. Both patients were confirmed to have measles through laboratory testing.

State and local public health authorities launched an outbreak investigation including contact tracing, symptom monitoring, and laboratory confirmation of clinical and wastewater samples. The CDPHE outbreak investigation identified five additional laboratory-confirmed measles cases with symptom onset during August 11–27 among 225 household and health care facility contacts of the first two identified patients. Among the seven patients, one had documentation of measles vaccination. Local health care providers were encouraged by state and local authorities to continue recommending measles vaccination and to review vaccine inventories to ensure adequate stock.

### Retrospective Identification of Likely Source Cases

In early October, CDPHE was notified by public health officials in state A that members of an ill Mesa County family had exposed residents of their state during a family gathering in early August. CDPHE identified this Mesa County family as the likely source of the Mesa County measles outbreak. Although three family members sought care for symptoms in late July and specimens were obtained for measles testing, the request was canceled when one of the family members received a coincidental positive test result for group A *Streptococcus*, because of a nationwide shortage of measles immunoglobulin M testing reagents. All three family members were unvaccinated. Before being identified, after travel to state A, the family had been exposed to measles in state B in mid-July and subsequently interacted with the first two recognized measles patients in Mesa County during July 25–26. The family cases were epidemiologically linked through the CDPHE outbreak investigation. This linkage explains both the initial local spread in Mesa County and the viral detections in the early August wastewater samples.

## Preliminary Conclusions and Actions

The detection of measles virus RNA in consecutive wastewater samples likely indicated ongoing community transmission, and the detection of a high concentration alerted the local public health agency to transmission before cases were reported. The first cases were reported ≤4 days of wastewater specimen collection, allowing dissemination of comprehensive messaging regarding both wastewater surveillance measles detections and clinical data to local health care providers. Timeliness is critical for wastewater surveillance to serve as an alert to local public health authorities. Ongoing monitoring of wastewater surveillance data by local public health agencies in Colorado can provide an early indication of community measles circulation and guide public health messaging regarding potential transmission, signs and symptoms of measles, recommendations for vaccination, and instructions for seeking care. Wastewater surveillance data complement surveillance for clinical cases, alerting local authorities and aiding resource allocation for outbreak investigations and containment (*2*,*5*).

## Footnotes

*
MeaslesSymptomsandComplications|Measles(Rubeola)|CDC

†
dPCR:ATechnologyReview|PMC

§ A measles viral concentration that is below the empirically established limit of detection. The limit of detection is defined as >1,200 gene copies per liter and ≥3 positive partitions per sample well. A low-level detection indicates that measles genetic material was likely present, but the concentration was too low to be reliably quantified with statistical certainty.
